# Exploring MPM-FLIM for diagnostics of porokeratosis – a pilot study *ex vivo*

**DOI:** 10.1364/BOE.558519

**Published:** 2025-05-05

**Authors:** Jeemol James, Rahime Inci, Noora Neittaanmäki, Despoina Kantere, Sirkku Peltonen, Marica B. Ericson

**Affiliations:** 1 University of Gothenburg, Biomedical photonics group, Department of Chemistry and Molecular Biology, Gothenburg, Sweden; 2 University of Gothenburg, Institute of Clinical Sciences, Department of Dermatology and Venereology, Gothenburg, Sweden; 3 Sahlgrenska University Hospital, Department of Dermatology and Venereology, Region Västra Götaland, Gothenburg, Sweden; 4 University of Gothenburg, Department of Laboratory Medicine, Institute of Biomedicine, Sahlgrenska Academy, Gothenburg, Sweden; 5 University of Helsinki and Helsinki University Hospital, Department of Dermatology and Allergology, Helsinki, Finland; 6 jeemol.james@gu.se; 7 marica.ericson@gu.se

## Abstract

Non-invasive diagnostic methods are essential for early detection and monitoring of skin diseases. This study evaluates multiphoton microscopy with fluorescence lifetime imaging (MPM-FLIM) as a diagnostic tool for porokeratosis. Skin biopsies were analysed using MPM-FLIM at 780 nm excitation wavelength targeting NADH and keratin fluorescence. Key morphological features were identified, such as the presence of cornoid lamella, confirmed by histopathology. Notably, keratin fluorescence (∼1500 ps) was found to dominate in porokeratotic samples, compared to NADH (∼450 ps) in normal skin. These findings support the potential of MPM-FLIM as a valuable diagnostic and research tool for rare skin disorders like porokeratosis.

## 1. Introduction

In the field of biomedical optics, Multiphoton laser scanning microscopy (MPM) has become an important non-invasive 3D imaging tool for biological specimens at subcellular resolution [1–3]. MPM offers improved imaging depth and reduced phototoxicity as compared to confocal microscopy. Through the simultaneous absorption of two photons, molecular excitation is achieved within a femtoliter volume of the sample, facilitated by femtosecond laser pulses operating in the near-infrared region.

Beyond fluorescence visualization, MPM can be combined with time resolved techniques. Recent studies explore label free imaging combined with fluorescence lifetime imaging microscopy (FLIM) based on time correlated single photon counting (TCSPC) [4–8]. FLIM utilizes the fluorescence lifetime of the fluorophores to construct high resolution contrast images [9,10]. In biological tissue samples, a FLIM image comprises fluorescence lifetime data originating from more than one fluorophore at each pixel. Thus, the fluorescence lifetime is extracted by fitting the fluorescence decay with multiexponential decay model. The combination of MPM-FLIM is explored as a powerful tool to map complex fluorophores distributions in biological samples given that the fluorescence lifetime is depending on the microenvironment of the imaging region [11–13]. Most MPM-FLIM studies primarily target the endogenous cellular fluorophores such as nicotinamide adenine dinucleotide (NADH) and flavin adenine dinucleotide (FAD) to monitor the cellular metabolism [14–17]. However, other cellular fluorophores like keratin, melanin, elastin, and collagen in tissues are also important [18,19]. In our earlier work, we have reported a significant spectral overlap of the emission from keratin and NADH when skin tissue is investigated [20,21]. Keratin is a structural protein which plays a vital function in maintaining the structural integrity of the skin as keratinocytes being the building block of epidermis in human skin [23,24]. This observation led us to further explore the relevance of fluorescence life-time signal originating from keratin in a biomedical context. Therefore, this study examines the adaptation of MPM-FLIM for the investigation of porokeratosis, a rare skin disorder in which keratinization play a crucial role [22].

Porokeratosis is a group of rare genetic skin disorders primarily caused by pathogenic variants in genes encoding enzymes involved in the mevalonate pathway [25–27]. One of the end products of this pathway is cholesterol, a crucial lipid component of the stratum corneum (SC), being the outermost layer of human skin formed through a process called keratinization. Lesions of porokeratosis are characterized by focal hyperkeratosis, which is abnormal keratinization at the border of the lesion. This focal hyperkeratosis produces a cornoid lamella, which is a distinct ridge-like hyperkeratotic border present in the SC. As the porokeratosis lesion enlarges, new cornoid lamellae form at its border. Correct diagnosis of porokeratosis is crucial for correct treatment, because it mimics several other skin conditions whose treatments are not applicable to porokeratosis. In addition, a recent epidemiological study identified porokeratosis as one of the most common genodermatoses in Sweden, being linked to a higher risk of skin cancer [28]. It was found that patients with porokeratosis experience a 1.8-4.3 times increased risk for skin cancer.

Clinical diagnosis of porokeratosis is performed through dermoscopy examination followed by skin biopsy and histopathology analysis. The presence of cornoid lamella is the characteristic diagnostic sign of porokeratosis. Skin biopsy and histopathological examination are referred to as a golden standard in the clinical diagnosis of skin diseases in general, as well as for porokeratosis. Since histopathology is invasive, time-consuming and labor-intensive process, there is a huge need for developing novel techniques that can operate directly in the clinical intervention rooms in a non-invasive fashion. In this context, the exploration of optical techniques such as MPM-FLIM has intensified over the years [8,29,30]. So called optical biopsy techniques, can provide patient comfort, early detection and monitoring. In relation to porokeratosis, optical biopsies can assist in early detection and thereby avoid malignant transformation. In addition to MPM-FLIM, other techniques are investigated. For example, reflectance confocal microscopy (RCM) and optical coherence tomography (OCT) are emerging technologies for similar purposes [29,31–33]. Recent studies have reported the application of RCM and OCT for the diagnostics of porokeratosis [22,34]. In our team, we have compared the efficacy of dermoscopy, RCM and MPM for diagnosing porokeratosis [35], with the conclusion that MPM offers better resolution, contrast and penetration depth as compared to RCM.

In the present study, we investigated the potential of MPM-FLIM for diagnosing porokeratosis through the *ex vivo* imaging of skin lesions obtained from patients. Imaging was conducted at a 780 nm excitation wavelength to visualize morphological features including the cornoid lamella and to correlate FLIM data with histological images. Changes in fluorescence lifetime data from different z levels were studied and compared with healthy skin tissue. To the best of our knowledge this study is the first ever attempt to visualise porokeratosis using *ex vivo* MPM-FLIM. We believe that cornoid lamella of porokeratosis serves as an interesting model of local aberrant keratinization, which broader implications for studying the keratin autofluorescence in various tissues.

## 2. Methods and materials

### 2.1. Porokeratosis skin biopsy and histopathology

Porokeratosis skin biopsies were obtained from porokeratosis patients from department of dermatology and venereology, Sahlgrenska university hospital, Gothenburg, Sweden as a part of ongoing study approved by Swedish ethical approval committee (Dnr 2021-02279). Porokeratosis biopsies of 4 mm size were obtained from six patients who are 18 years or older with consent. Each biopsy was cut into two parts, one of the parts was used for MPM-FLIM imaging and the other part was utilized for histopathology examination. Detailed descriptions of the biopsies and patient demography are included in the Supplement 1 (Table S1). As a control, frozen healthy skin lesion obtained from breast reduction surgery was used for MPM-FLIM imaging.

### 2.2. MPM-FLIM imaging

An experimental inverted MPM setup equipped with fs-pulsed tunable Ti-Sa laser (MaiTai DeepSee, Newport Spectra-Physics) was used for MPM-FLIM imaging as explained in the previous work [36]. Porokeratosis skin biopsies were placed on a custom-built imaging chamber with surface of the skin facing the coverslip when imaged at 780 nm wavelength. Laser power at the sample was kept approximately around 20 mW controlled by an external acousto-optic modulator (MT110-B50A1.5-IR-Hk, AA Opto-Electronic). This power level is below the safe limit suggested by IEC (international electrotechnical commission)60601-2-22 standard for basic safety of laser used in diagnostic purpose [37]. A long working distance water immersion objective lens (W Plan apochromat 20x/1.0 DIC VIS-IR, Carl Zeiss) and another water immersion objective lens (C achroplan NIR, 40x/0.8 W, Carl Zeiss) were employed. To enable FLIM acquisition, two GaAsP detectors (H7422P-40 MOD, Hamamatsu), interfaced with two TCSPC modules (SPC 150, Becker and Hickel) were utilized in the emission pathway. A dichroic mirror (509 nm cut off, Semrock Inc) combined with two filters 445/60 nm and 580/150 nm (Semrock Inc, Brightline) was utilized to enable two spectral channels separately for detecting NADH and keratin emission respectively. FLIM images were recorded using SPCM64 software (Becker and Hickel) at 512 × 512 pixels, a pixel dwell time of 1.8 µs, scanning speed 0.616 per frame and 60 s image acquisition time. MPM-FLIM investigations were performed and images acquired from a total of 2-3 different locations from each tissue biopsy.

### 2.3. FLIM data analysis

MPM-FLIM images were processed and analysed by using SPCImage software (V9.88, Becker and Hickel). An intensity threshold value specific to each image was applied to eliminate the dark background regions from FLIM analysis. The instrument response function (IRF) was calculated using the ‘auto IRF’ option in the software where the full width half maximum was approximately 250 ps. To identify the best data fitting model for FLIM analysis, all the pixels in the whole field of view from each image were binned.

A triple exponential decay model with maximum likelihood estimation (MLE) fit model was chosen for FLIM analysis of porokeratosis skin biopsy,

(1) f(t)=f(t)=a0+a1e−
t/τ
1+a2e−
t/τ
2+a3e−
t/τ
3

where τ
1 correspond to short lifetime and τ
2 , τ
3 represent long lifetime components. The pre-exponential factor $a_{1}$ describes amplitude component of the fast fluorescence decay and $a_{2}$ , $a_{3}$ correspond to the slow fluorescence decay signal. As a first step, a triple exponential decay model was applied by binning all the pixel in whole image and average value of τ
1 and τ
2 were calculated. The FLIM analysis was repeated by fixing τ
1 and τ
2 to their average values from the initial fit. This fit was applied to pixel wise decay by allowing τ
3 and the pre-exponential factors to fit freely by keeping the $x^{2}$ value to approximately around 1 as a goodness of fit. Histograms and figures were plotted using MATLAB (v2024b, MathWorks Inc). The data were analysed for every different location in each sample, yielding equivalent results.

## 3. Results

**Fig. 1.**
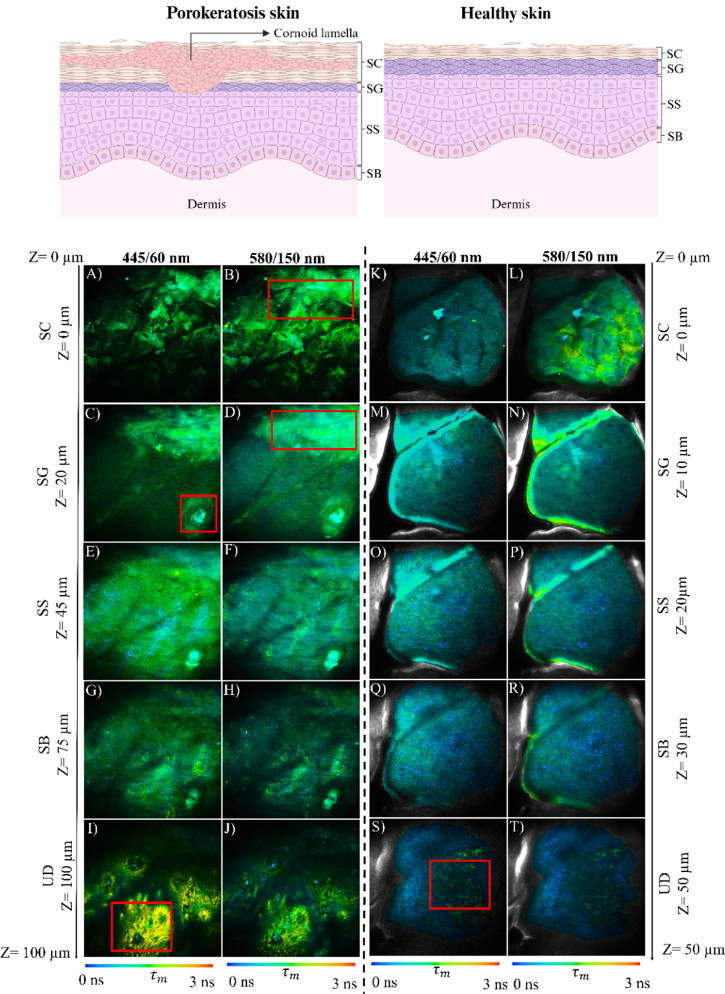
Schematic overview (top panel, *BioRender. James, J. (2024) BioRender.com/u30y475*) and MPM-FLIM data from porokeratosis skin lesion sample no GENEMIC09 (A-J) and healthy skin (K-T) acquired *ex vivo* using 780 nm excitation. The corresponding epidermal layers stratum corneum (SC), stratum granulosum (SG), stratum spinosum (SS), stratum basale (SB) and upper dermis (UD) are denoted at different tissue depth (i.e. Z-levels). Highlighted region of interest (red boxes) represent part of cornoid lamella (B,D), hair follicle in SG (C), part of elastin and collagen network in UD (I,S). Field of view of images (A-J) ∼ 350 × 350 µm^2^ and (K-T) ∼ 250 × 250 µm^2^. False color scale represents fluorescence lifetime ranging from 0-3 ns using 256-time channels.

*Ex vivo* MPM-FLIM images acquired from one of the tissue samples, sample no GENEMIC09, are presented in Fig. 1, together with reference data from control healthy skin tissue and a schematic illustration of the expected morphological features observed in porokeratotic and normal skin. The MPM-FLIM data are presented for both spectral channels (450/60 nm and 580/150 nm), and excitation wavelength set to 780 nm. As demonstrated in the Fig. 1, the corresponding Z-levels for the different epidermal layers differ between the parakeratotic sample (Fig. 1(A)-(J)) and the normal skin control (Fig. 1 K-T). Particularly, the SC layer of porokeratosis skin sample was thicker than control skin, 20 µm vs 10 µm, respectively. This observation was consistent across all the investigated porokeratosis lesions, see Table S2 Supplement 1.

When comparing the fluorescence lifetime data acquired for porokeratosis and control healthy skin samples, it is observed that, the average fluorescence lifetimes for the porokeratosis sample are similar across both spectral channels and throughout the epidermal layers (Fig. 1(A)-(J)). The average fluorescence lifetime was around 1500 ps and represented by green color scale. For comparison, the observed fluorescence lifetimes in the control sample (Fig. 1(K)-(T)) were dominated by shorter lifetime values closer to 450 ps, demonstrating a larger variability between the spectral channels, as well as within the different tissue layers. This result suggests that the control sample (Fig. 1 K-T) is primarily dominated by NADH fluorescence, while the signal acquired from the porokeratosis lesion (Fig. 1(A)-(J)) is dominated by autofluorescence most likely originating from keratin [38,39]. A shift from longer lifetime in green towards short lifetime NADH fluorescence in blue is observed for the control sample in the transition from stratum corneum (SC) (Fig. 1(L)) to stratum granulosum (SG) (Fig. 1(N)) in the red shifted spectral channel (i.e. 580/150 nm). This is likely due to the increase in keratin signal during cellular differentiation and formation of the cornified layer as observed in SC as a sign of normal keratinization. Flat sheet-like cells with distinct cell boundaries corresponding to the morphology of corneocytes were found in the SC layer of the porokeratosis sample as highlighted in the region of interest in Fig 1(B) (red rectangle region of interest). This cluster of cells dominated by keratin fluorescence extends into the SG region (Fig. 1(D)). This finding further supports our previous work [20] demonstrating the importance of considering the effect of keratin autofluorescence when applying MPM-FLIM for the investigation of skin samples. (See Supplement 1 Fig S2 and S3, for more detailed analysis of fluorescence lifetime data)

**Fig. 2.**
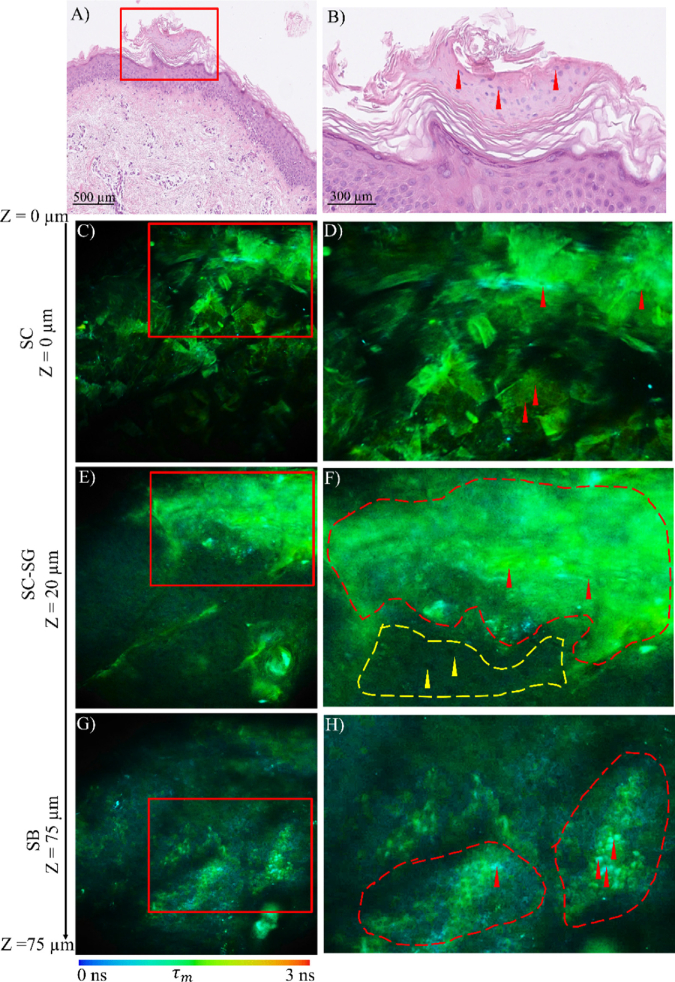
Histopathologic images (A, B) and MPM-FLIM images (C-H) from porokeratosis skin lesion sample no GENMIC09. The MPM-FLIM images equal the data in Fig. 1 for spectral channel 580/150 nm, corresponding to SC (C, D), SC-SG (E, F) and SB (G, H) at two different zoom levels. Highlighted region of interest (red box) represents cornoid lamella in histological image (A), SC (C) and SG (E) in MPM-FLIM image and dermal papillae in SB (G). Field of view MPM-FLIM images are ∼ 350 × 350 µm^2^ (C, E, G) and zoomed in areas marked with red rectangle region of interest ∼ 230 × 160 µm^2^ (D, F, H). False color scale represents fluorescence lifetime ranging from 0-3 ns using 256-time channels.

One of the most significant histopathologic features of porokeratosis is the presence of a so called cornoid lamella extending from stratum spinosum (SS) to SC (Fig. 1, top panel). The cornoid lamella consists of a dense layer of hyperkeratosis resulting from focal abnormal keratinocyte maturation [25,40]. This abnormal maturation leads to thickening of the SC, a condition known as hyperkeratosis, and the presence of nucleated cells in the SC- parakeratotic cells located on top of the cornoid lamella. Furthermore, the SG layer is absent or thinned under the cornoid lamella. We have recently demonstrated that MPM is superior to confocal reflectance microscopy in demonstrating the presence of cornoid lamella [35]. In the present study we extend the investigation to explore the additional information acquired with FLIM. A cluster of highly fluorescent parakeratotic cells, observed in bright green, extends down to 20 µm, still visible in the layer expected to be SG (highlighted red rectangle region Fig. 1(B), (D)). This signal is interpreted to correspond to the cornoid lamella. A more detailed comparison between tissue morphology observed using MPM-FLIM and histopathology focusing on the cornoid lamella will follow (see Fig. 2).

Additional morphological features observed include a hair follicle which was visible in the SG layer (Fig. 1(C), red region of interest). In porokeratosis, the MPM-FLIM demonstrate a more uniform fluorescence lifetime distribution acquired from stratum spinosum (SS) (Fig. 1(E)-(F)) compared to the normal skin (Fig. 1(O)-(P)) where presence of keratinocytes and cell nuclei was discerned. Furthermore, the stratum basale (SB) differs significantly between lesion (Fig. 1(G)-(H)) and normal skin (Fig. 1(Q)-(R)). Features exhibiting longer fluorescence lifetime values approximately around 2000 ps appears in the transition layer corresponding to upper dermis (UD) discerned in yellow-orange color (Fig. 1(I)-(J), red rectangle region of interest) and as green shifted in control (Fig. 1(S)-(T)). This shift in fluorescence lifetime is expected as elastin and collagen network are known to exhibit fluorescence lifetimes around 2000 ps [19], and therefore serves as an important reference layer when investigating skin tissue using MPM-FLIM.

In Fig. 2, a more detailed analysis of the porokeratosis morphology observed using MPM-FLIM (Fig. 2(C)-(H)) is presented in comparison with corresponding histopathologic examination (Fig. 2(A)-(B)). The MPM-FLIM images match the images in Fig. 1 (580/150 nm spectral channel) at a larger magnification (Fig. 2(C), (E), (G)) with additional zoomed in areas (Fig. 2(D), (F), (H)) corresponding to SC, SG and SB. The confirmed histopathological presence of cornoid lamella is highlighted in SC (Fig 2(A), (red rectangle)), further supported by presence of pillar of nucleated parakeratotic keratinocytes (Fig 2(B) (red arrows)). Histopathology shows a general thickening of SC and thinning of SG layer (Fig. 2(A)). In the MPM-FLIM image of SC (Fig 2(C)), a compact layer of corneocytes was observed, exhibiting fluorescence lifetime values of approximately 1500 ps, characteristic of keratin fluorescence. In the zoomed in image (Fig 2(D)) the corneocytes are more clearly discerned and presence of nucleated cells is highlighted (red arrows).

The signatory thickening of SC is effectively detected by MPM-FLIM as tissue morphology corresponding to SG appeared at a depth of 20 µm (Fig. 2(E)), which deviates from the healthy control (Fig. 1(M)-(N)) appearing at 10 µm. The zoomed in image (Fig. 2(F)), focusing on the cornoid lamella, demonstrate two morphologically different regions part of cornoid lamella in SC layer (red region of interest) and SG layer (yellow region of interest). The region corresponding to the cornoid lamella is represented by a cluster of nucleated parakeratotic cells exhibiting bright keratin autofluorescence (Fig 2(F), (red region and arrows)). In addition, a region with less fluorescent granular keratinocytes with dark nuclei and diffusing cytoplasm was observed in SG layer (Fig 2(F), (yellow region and arrows)). This further supports the conclusion that MPM-FLIM can be employed to visualize thickening of SC and demonstrate presence of cornoid lamella in porokeratotic skin lesions, as discussed earlier.

In the SB (Fig 2(G)), the observed fluorescence lifetime values shift towards shorter lifetimes (less than 1000 ps) appearing in blue using the applied color-scale. This shift is anticipated as the presence of NADH is expected to be more pronounced in the deeper epidermal layers as cell metabolism is higher, in agreement with our previous work [20,21]. In addition, cells with distinct shorter lifetimes (∼300 ps) and lack of cell nuclei were observed (Fig 2(H), (red arrows)). As these cells appear close to regions resembling vicinity to dermal papillae, it is plausible that these short lifetime cells correspond to melanocytes, or keratinocytes with high melanin content, as melanin is known to exhibit a short fluorescence lifetime (∼280 ps) [41,19].

**Fig. 3.**
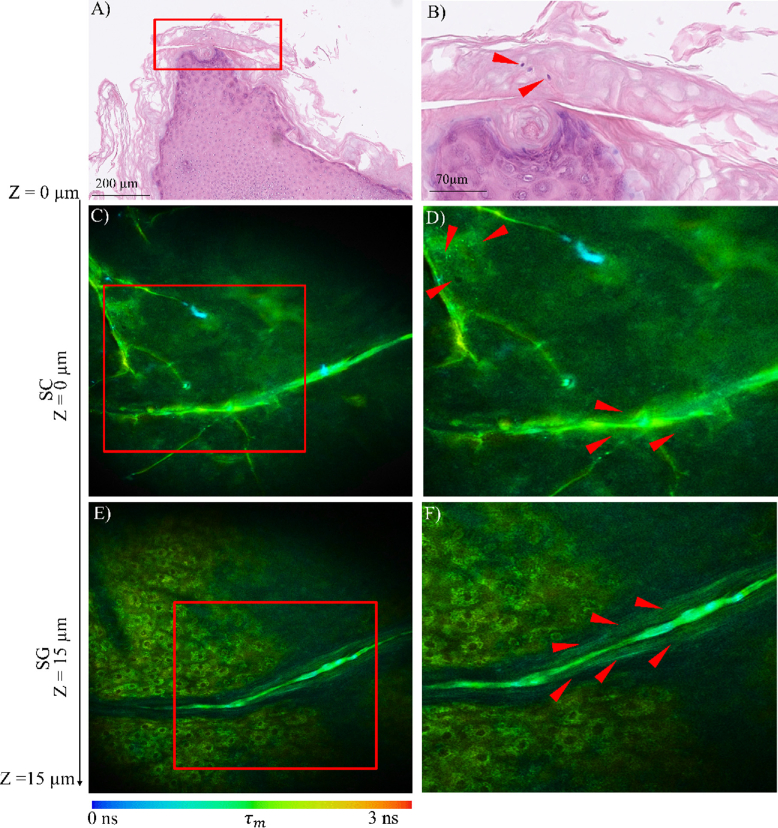
Histopathologic images (A, B) and MPM-FLIM images (C-F) from porokeratosis skin lesion sample no GENMIC08. The MPM-FLIM images are from spectral channel 580/150 nm and correspond to SC (C, D), and SG (E, F) at two different zoom levels. Highlighted region of interest (red box) represents cornoid lamella in histological image (A), part of cornoid lamella in SC (C) and SG (E) in MPM-FLIM image). Field of view MPM-FLIM images is ∼ 350 × 350 µm^2^ (C, E) and zoomed in areas marked with red rectangle region of interest ∼220 × 230 µm^2^ (D, F). False color scale represents fluorescence lifetime ranging from 0-3 ns using 256-time channels.

A similar detailed comparison between MPM-FLIM and histopathology is presented for an additional sample biopsy (sample no GENMIC08) in Fig. 3. As seen in the histopathological images (Fig. 3(A)-(B)), a column of hyperkeratotic SC with a cornoid lamella was confirmed (red rectangular region of interest), with presence of parakeratotic cells (Fig. 3(B), (red arrows)). In the MPM-FLIM images (Fig. 3(C)-(F)), the autofluorescence from keratin is dominant with an average fluorescence lifetime ∼ 1500 ps. Complete MPM-FLIM images of the entire Z-stack from the lesion is included in Supplement 1 (Fig.S1). In the SC, the presence of cornoid lamella is observed as bright fluorescent green lines (Fig 3(C)-(D), (red arrows)). Interestingly, additional bright fluorescent spots were observed as scattered green bursts (Fig 3(D), (red arrows)). Large granular keratinocytes with visible nuclei and cytoplasm were present in SG approximately 15 µm below SC (Fig. 3(E)). Also seen from the image, is a continuation of the cornoid lamella observed as a bright green line, which at higher magnification shows characteristic elongated cornified structures (Fig 3(F) (red arrows)). These elongated structures most likely corresponds to flattened granular keratinocytes as the cornoid lamella extends from SC to SG, as confirmed by histopathology.

Altogether the morphological features observed in MPM-FLIM are comparable to the findings of histopathological analysis when investigating porokeratosis. Furthermore, MPM-FLIM conveys spectroscopic information by which the dominance from keratin over NADH offering valuable information about the degree of keratosis. Therefore, these results altogether support the conclusion that MPM-FLIM can be employed as a valuable tool for studying and diagnosing porokeratosis.

## 4. Discussion and conclusion

This study demonstrates the efficacy of MPM-FLIM as a diagnostic tool for porokeratosis skin lesions. Diagnosing porokeratosis is crucial, as certain types of porokeratosis carry the risk of malignant transformation and can be misdiagnosed as other skin disorder. MPM-FLIM effectively visualizes key morphological and fluorescence lifetime differences, including the thickened SC and the presence of the cornoid lamella, both characterized by keratin autofluorescence. Additionally, layer-specific changes, and presence of dermal papillae in SS and SB, were observed in the affected tissues. Moreover, MPM-FLIM images of healthy skin lesion demonstrated the typical morphological and cellular structures as described in literature [6,42].

From a histological perspective the presence of cornoid lamella with parakeratotic cells in SC is the standard diagnostic criteria for porokeratosis. The most significant finding observed by MPM-FLIM is the presence of cornoid lamella and the appearance of nucleated parakeratotic cells in SC. Additionally, thickening of the SC layer (∼ 15-20 µm) is observed in porokeratosis skin lesions as compared to the healthy control sample. MPM-FLIM data obtained from porokeratosis skin lesion were dominated by the abundance of keratin signal seen as green color (∼1500 ps) whereas healthy skin was dominated by NADH signal (∼ 450 ps) in blue color. This difference is likely attributed to abnormal keratinization caused by the hyperkeratosis in porokeratosis lesions and signatory for the presence of cornoid lamella. Keratin fluorescence is frequently overlooked in many studies, where the primary emphasis is placed on NADH fluorescence. In alignment with our previous studies [20], this pilot report confirms the significance of keratin fluorescence in MPM-FLIM studies focusing on keratin related skin disorders.

The research to date has tended to focus on applying MPM-FLIM for visualization and monitoring cellular metabolism and not explored the diagnostic potential of MPM-FLIM for skin disorders [5,43,44]. In our previous study, we have investigated the possibility of MPM-FLIM to image metastasis in human sentinel lymph node targeting to diagnose melanoma [45]. And we realized, challenges such as limited access to clinical bio-samples, stringent ethical approvals, lack of interdisciplinary opportunities, requirement of training and expertise on the optical techniques must be addressed to advance MPM-FLIM as a reliable optical diagnostic tool. In this study, MPM-FLIM is currently limited to visualizing a small, localized area of a biopsy. In contrast, histopathological analysis remains the gold standard for diagnosis, offering a comprehensive overview of the entire tissue sample. Advancements in imaging technology have the potential to enhance the diagnostic capabilities of MPM-FLIM, allowing it to serve as a promising complementary tool to histopathology. To establish MPM-FLIM as a reliable diagnostic method for rare skin disorders, further validation studies and continued research across a wide range of tissue types and dermatological conditions are essential. This could contribute to the development of a comprehensive directory of MPM-FLIM characteristics associated with various skin disorders, highlighting how these imaging features correlate with corresponding histological findings. In addition, fundamental studies focusing on the fluorescence of different isoforms of keratin and how it can be related to other keratinization skin disorders need to be performed as keratin plays a key role in major skin disorders [46–48]. The possibility of combining MPM-FLIM with other imaging modalities such as optical coherence tomography (OCT) needs to be investigated to facilitate the development of *in vivo* diagnostic tools for skin disorders. Therefore, this pilot study set to demonstrate the potential of MPM-FLIM to visualize porokeratosis and understand the changes in the keratin lifetime data at different skin layers in *ex-vivo* skin lesions obtained from porokeratosis patients. In addition, MPM-FLIM results are compared with histopathological observations confirming the possibility and feasibility of using MPM-FLIM as a complementary technique for diagnosis.

To conclude, the experimental exploration presented here aims to contribute to the ongoing development of MPM-FLIM as a non-invasive diagnostic tool for skin related diseases and contributes to the better understanding of keratin representation when interpreting autofluorescence in skin. By offering diagnostic potential, MPM-FLIM highlights its significant ability for developing non-invasive evaluation of keratin-related skin disorders and broader clinical applications in dermatology in the future. Taken together, advancements in microscopy, imaging techniques, and the integration of artificial intelligence support the future clinical translation of *in-vivo* MPM-FLIM for diagnosing skin disorders.

## Supplemental information

Supplement 1 supplementary document contains all the supplemental figures and tables mentioned in the manuscript https://doi.org/10.6084/m9.figshare.28722299

## Data Availability

The data presented in this study are available from the corresponding author upon reasonable request.

## Supplemental document

See Supplement 1 for supporting content.
