# Supplementary material for: Exploring MPM-FLIM for diagnostics of porokeratosis – a pilot study ex vivo
Source: Biomed Opt Express. 2025 May 5;16(6):2243–53. doi: 10.1364/BOE.558519 (PMC12945498; doi:10.1364/BOE.558519)
Supplement: Supplementary file 1 [file boe-16-6-2243-s001.pdf]

Supplemental Document

Biomedical Optics EXPRESS

Exploring MPM-FLIM for diagnostics of porokeratosis – a pilot study *ex vivo*: supplement

JEEMOL JAMES,[1,6] RAHIME INCI,[2,3] NOORA NEITTAANMÄKI,[4] DESPOINA KANTERE,[2,3] SIRKKU PELTONEN,[2,3,5] AND MARICA B. ERICSON[1,7]

[1] *University of Gothenburg, Biomedical photonics group, Department of Chemistry and Molecular Biology, Gothenburg, Sweden*
[2] *University of Gothenburg, Institute of Clinical Sciences, Department of Dermatology and Venereology, Gothenburg, Sweden*
[3] *Sahlgrenska University Hospital, Department of Dermatology and Venereology, Region Västra Götaland, Gothenburg, Sweden*
[4] *University of Gothenburg, Department of Laboratory Medicine, Institute of Biomedicine, Sahlgrenska Academy, Gothenburg, Sweden*
[5] *University of Helsinki and Helsinki University Hospital, Department of Dermatology and Allergology, Helsinki, Finland*
[6] *jeemol.james@gu.se*
[7] *marica.ericson@gu.se*

This supplement published with Optica Publishing Group on 5 May 2025 by The Authors under the terms of the Creative Commons Attribution 4.0 License in the format provided by the authors and unedited. Further distribution of this work must maintain attribution to the author(s) and the published article's title, journal citation, and DOI.

Supplement DOI: https://doi.org/10.6084/m9.figshare.28722299

Parent Article DOI: https://doi.org/10.1364/BOE.558519

Supplementary data

Exploring MPM-FLIM for diagnostics of porokeratosis – A pilot study *ex vivo*

Jeemol James[1,*], Rahime Inci[2,3], Noora Neittaanmäki[4], Despoina Kantere[2,3], Sirkku Peltonen[2,3,5] and Marica B. Ericson[1,*]

[1]*University of Gothenburg, Biomedical photonics group, Department of Chemistry and Molecular Biology, Gothenburg, Sweden*

[2]*University of Gothenburg, Institute of Clinical Sciences, Department of Dermatology and Venereology, Gothenburg, Sweden*

[3]*Sahlgrenska University Hospital, Department of Dermatology and Venereology, Region Västra Götaland, Gothenburg, Sweden*

[4]*University of Gothenburg, Department of Laboratory Medicine, Institute of Biomedicine, Sahlgrenska Academy, Gothenburg, Sweden*

[5] *University of Helsinki and Helsinki University Hospital, Department of Dermatology and Allergology, Helsinki, Finland*

[*]*Corresponding authors, e-mail:* jeemol.james@gu.se, marica.ericson@gu.se

Table S1: Deatiled information about the demography of the porokeratosis patients included in the study

Patient No	Year	Age	Gender	Disease duration	Location
GENMIC04	2022	32	Female	31 years	Left leg
GENMIC05	2022	80	Female	50 years	Left arm
GENMIC08	2022	73	Female	20 years	Right leg
GENMIC09	2022	53	Male	25 years	Back
GENMIC10	2022	46	Female	5 years	Left leg
GENMIC11	2023	64	Female	30 years	Left leg

Table S2: Imaging depth (Z stack) and thickness of SC layer obtained from MPM-FLIM iamges of of porokeratosis skin biopsies

Pateint No	Total imaging depth of the Z stack	Thickness of startum corneum
GENMIC 04	100 μm	15μm
GENMIC05	MPM-FLIM could not be done as the sample was damaged	
GENMIC08	100 μm	20 μm
GENMIC09	100 μm	20 μm
GENMIC10	105 μm	20 μm
GENMIC11	115 μm	20 μm
Healthy skin	50 μm	10μm

Figure S1 shows ex *vivo* MPM-FLIM images of porokeratosis skin biopsy (sample no GENEMIC08) obtained from different skin layers obtained from two different spectral channels (450/60 nm and 580/150 nm) when excited at 780 nm wavelength. As illustrated in Fig S1 MPM-FLIM images of each porokeratosis skin biopsies were taken from different epidermal skin layers having different Z levels, starting from SC (Z= 0 μm) until upper dermis layer (UD, Z= 100 μm). Comparing the FLIM images from two spectral channels, autofluorescence signal strength is uniform in both the channels and dominated by keratin as represented in green color corresponding to average fluorescence lifetime 1500 ps. In SC layer, possible part of cornoid lamella as seen as bright green lines as highlighted (red rectangle in Fig S1.B) in the region of interest was present. Large granular keratinocytes were visible with visible nuclei and cytoplasm were spotted in SG layer lying 20 μm below SC (highlighted yellow rectangle in Fig S1.D). Moreover, continuation of cornoid lamella is seen as bright green line as highlighted in SG layer (red rectangle in Fig S1.D). The average fluorescence lifetime, τ_m in SC is around 1500 ps in the spectral channel 580/150 nm which is like the lifetime value of keratin. In SS layer, more uniform distribution of keratinocytes was visible. A group of scattered cells possibly corresponding to the part of dermal papilla were found (red rectangle region of interest) in the SB layer (Fig S1.F). Elastin and collagen network exhibiting long lifetime values approximately around 2000 ps as seen as yellow-orange color started to emerge in SB and became visible in UD as highlighted (red rectangle region of interest) in Fig S1.I. MPM-FLIM was able to obtain 3D images of porokeratosis skin biopsy deep down to a depth of 100 μm from the SC level and demonstrated morphological features including cornoid lamella.

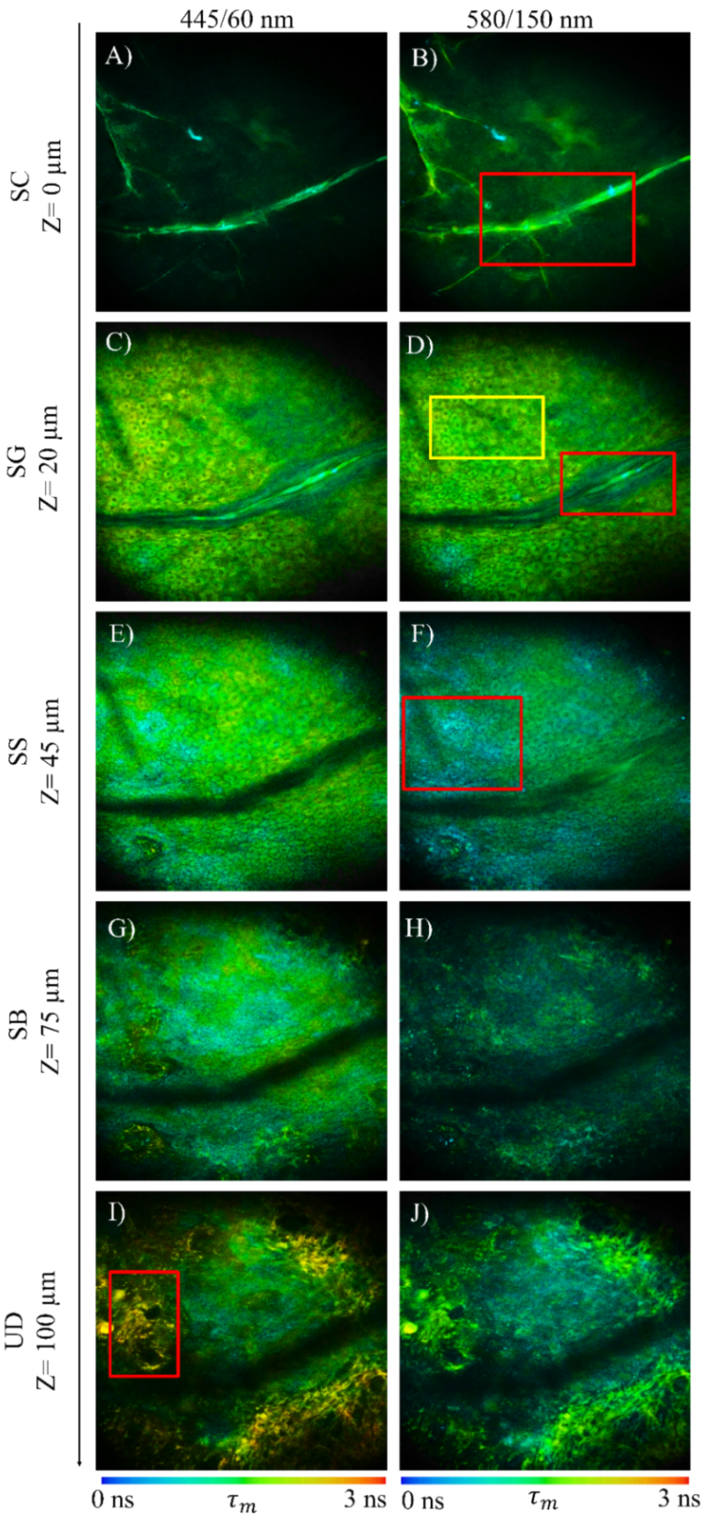

Figure S1: MPM-FLIM images of porokeratosis skin biopsy (sample no GENMIC08) *ex vivo* including a hyperkeratotic cornoid lamella excited at 780 nm obtained from 445/60 nm and 580/150 nm spectral channels. MPM-FLIM images obtained from different layers comprising stratum corneum (SC), stratum granulosum (SG), stratum spinosum (SP), stratum basale (SB) and upper dermis (UD). Highlighted region of interests corresponds to part of cornoid lamella (red rectangle) in SC (B), granular layer keratinocytes (yellow rectangle) and part of cornoid lamella in SG (D), possible group of melanocytes (red rectangle) in SS (F) and collagen, elastin network (red rectangle) in UD (I). Field of view ~ 350 x 350 µm^2. False color scale ranging from 0-3 ns from 256-time channels.

Figure S2 shows an overview of distribution of average fluorescence lifetime histograms corresponding to different epidermal layers of (SC,SG,SS,SB and UD) porokeratosis biopsies (A-D) and healthy control skin (E-F) obtained from 445/60 nm and 580/150 nm spectral channels. As seen in the figure, the fluorescence average lifetime corresponds to SC layer is around 1500 ps in 580/150 nm spectral channel for porokeratosis skin lesions (B,D) which confirms the presence of keratin. In addition to that the average fluorescence lifetime corresponds to other epidermal layers (SG,SS,SB and UD) is above 500 ps for porokeratosis skin lesions in both the spectral channels. Whereas average fluorescence lifetime corresponds to other epidermal layers of healthy control skin lesions is observed around 500 ps. This shows the dominance of NADH signal over keratin in normal healthy skin as compared to keratin rich porokeratosis lesions.

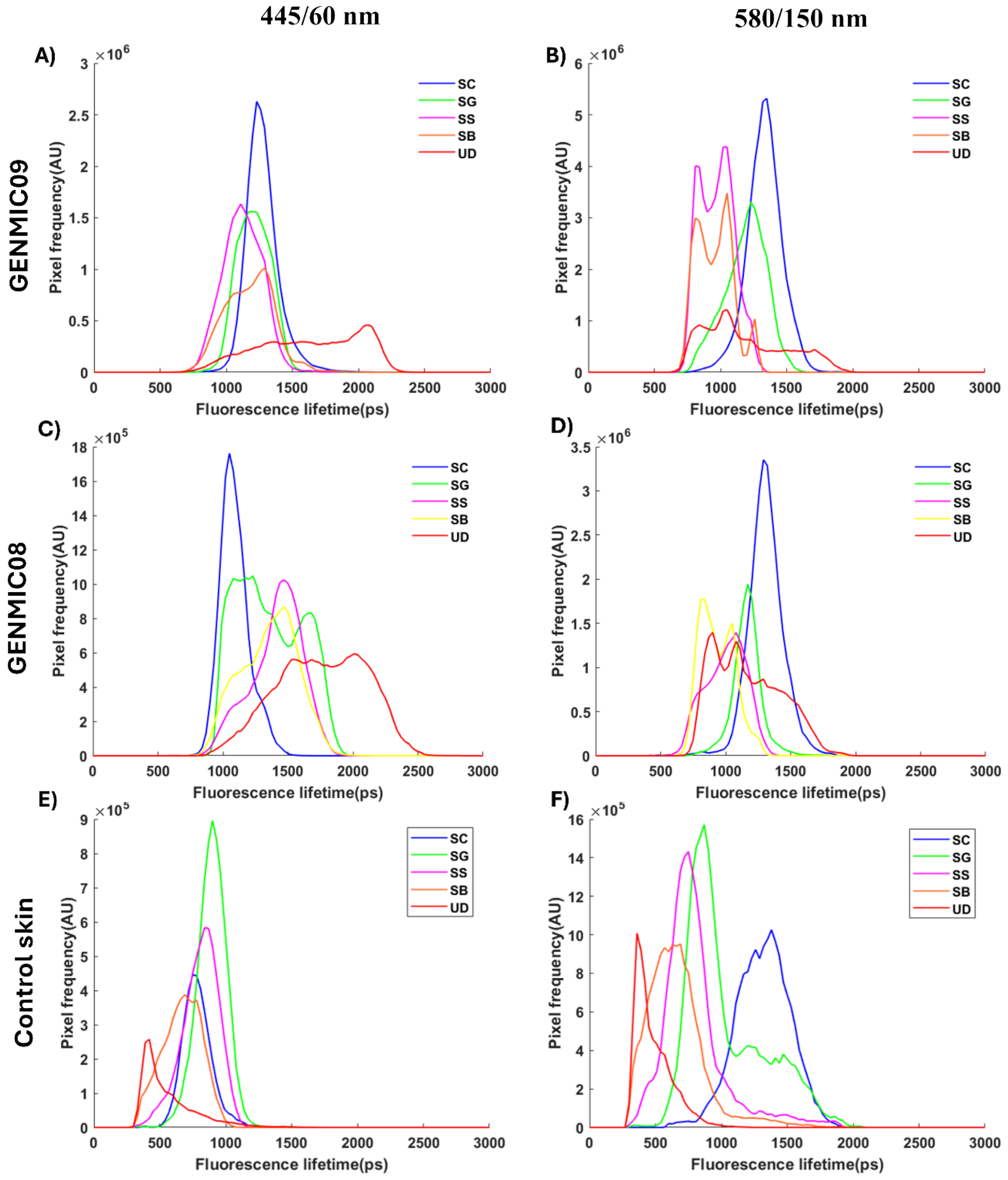

Figure S2: Fluorescence lifetime histogram distribution obtained from porokeratosis skin biopsies (A-D) and healthy control skin (E-F) obtained from two different spectral channels 445/60 nm and 580/150 nm corresponding to different epidermal layers (SC,SG,SS,SB and UD).

Figure S3 shows an overview of distribution of pre-exponential factors corresponds to short lifetime component (450 nm) a1% from 445/ 60 nm spectral channel and long lifetime component (1500 ps) a2% from 580/150 nm spectral channel obtained from different epidermal layers (SC,SG,SS, SB and UD) of porokeratosis skin biopsies (A-D) and healthy control skin (E-F). In healthy control skin, a1% (E) corresponds to NADH contribution in 445/60 nm is increasing (from SC till UD layers) as compared to abnormal trend (A,C) in porokeratosis lesions. This abnormality is probably due to the complex distribution of keratin and NADH distribution in porokeratosis skin lesions. A2% (F) corresponds to keratin distribution in healthy control skin shows decreasing trend when imaging from SC until UD in 580/150 nm channel. In porokeratosis skin lesions, a2% shows similar decreasing trend from SC until SB and shows an increased contribution in UD (B,D). The increase in the contribution of a2% in UD is due to the presence of long lifetime components of elastin and collagen.

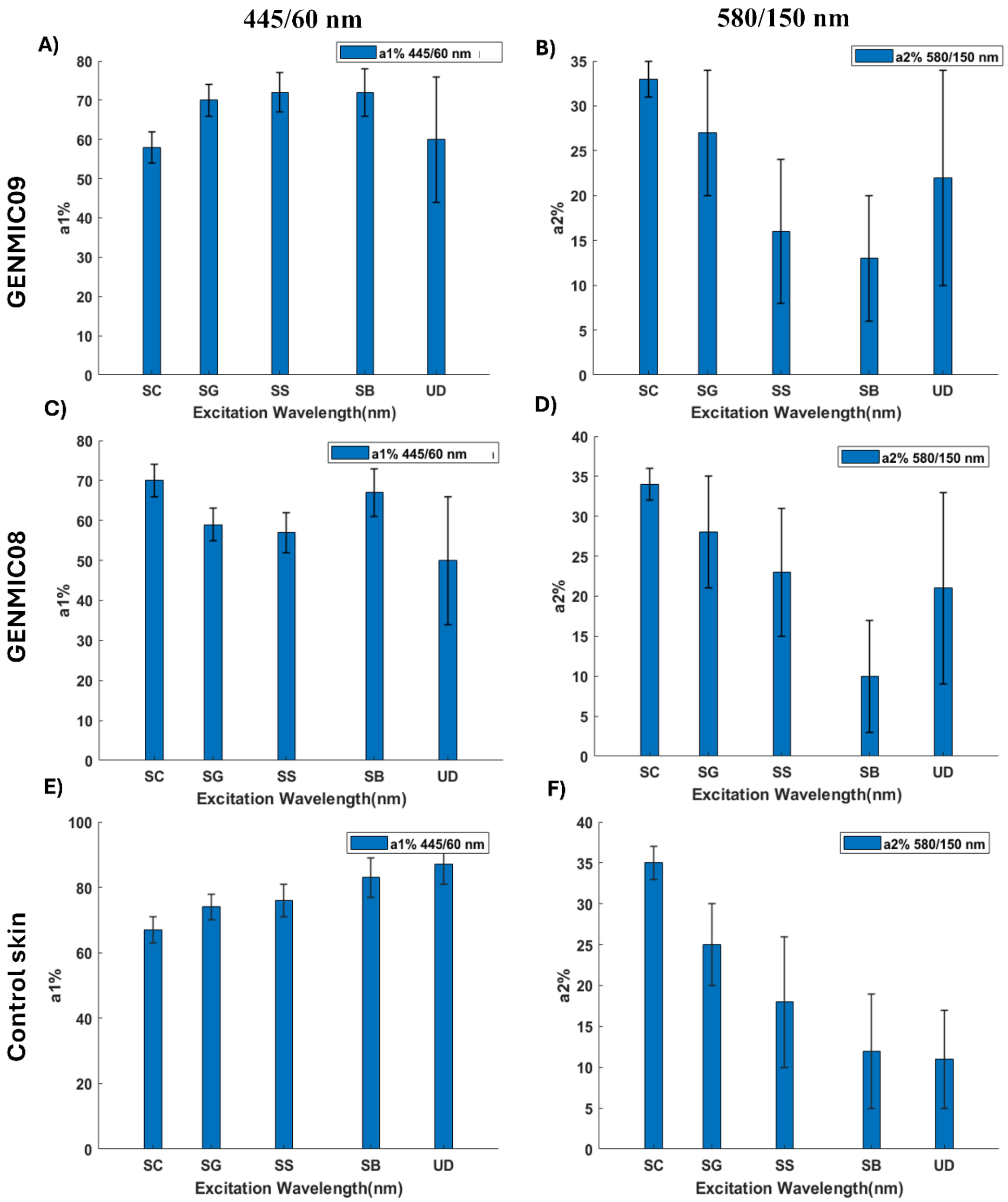

Figure S3: Distribution of pre-exponential factors corresponds to short lifetime component 450 nm, a1% from 445/ 60 nm spectral channel and long lifetime component a2% from 580/150 nm spectral channel obtained from different epidermal layers (SC,SG,SS, SB and UD) of porokeratosis skin biopsies (A-D) and healthy control skin (E-F).
